# Basic Amino Acid Residues of Human Eosinophil Derived Neurotoxin Essential for Glycosaminoglycan Binding

**DOI:** 10.3390/ijms140919067

**Published:** 2013-09-16

**Authors:** Ta-Jen Hung, Wei-Tang Chang, Noboru Tomiya, Yuan-Chuan Lee, Hao-Teng Chang, Chien-Jung Chen, Ping-Hsueh Kuo, Tan-chi Fan, Margaret Dah-Tsyr Chang

**Affiliations:** 1 Institute of Molecular and Cellular Biology, National Tsing Hua University, Hsinchu 300, Taiwan; E-Mails: d9680804@oz.nthu.edu.tw (T.-J.H.); extraby123@nhri.org.tw (W.-T.C.); ylee7@jhu.edu (Y.-C.L.); iverson13179@hotmail.com (C.-J.C.); s9980584@m99.nthu.edu.tw (P.-H.K.); 2 Department of Biology, Johns Hopkins University, Baltimore, MD 21218, USA; E-Mail: ntomiya1@jhu.edu; 3 Graduate Institute of Basic Medical Science, China Medical University, Taichung 404, Taiwan; E-Mail: htchang@mail.cmu.edu.tw; 4 Stem Cell and Translational Cancer Research Center, Chang Gung Memorial Hospital at Linkou, Taoyuan 333, Taiwan; E-Mail: tcf@gate.sinica.edu.tw; 5 Department of Medical Science, National Tsing Hua University, Hsinchu 300, Taiwan

**Keywords:** eosinophil derived neurotoxin, heparin, glycosaminoglycan, heparin binding region

## Abstract

Human eosinophil derived neurotoxin (EDN), a granule protein secreted by activated eosinophils, is a biomarker for asthma in children. EDN belongs to the human RNase A superfamily possessing both ribonucleolytic and antiviral activities. EDN interacts with heparin oligosaccharides and heparin sulfate proteoglycans on bronchial epithelial Beas-2B cells. In this study, we demonstrate that the binding of EDN to cells requires cell surface glycosaminoglycans (GAGs), and the binding strength between EDN and GAGs depends on the sulfation levels of GAGs. Furthermore, *in silico* computer modeling and *in vitro* binding assays suggest critical roles for the following basic amino acids located within heparin binding regions (HBRs) of EDN ^34^QRRCKN^39^ (HBR1), ^65^NKTRKN^70^ (HBR2), and ^113^NRDQRRD^119^ (HBR3) and in particular Arg^35^, Arg^36^, and Arg^38^ within HBR1, and Arg^114^ and Arg^117^ within HBR3. Our data suggest that sulfated GAGs play a major role in EDN binding, which in turn may be related to the cellular effects of EDN.

## 1. Introduction

Human eosinophil derived neurotoxin (EDN) is a basic protein (pI 8.9) normally stored in cytoplasmic granules and secreted by activated eosinophilic leukocytes [1]. It is also found in basophils, neutrophils, mononuclear cells and organs associated with these types of white blood cells [2–4]. EDN was initially discovered as a neurotoxin with selective killing effects on cerebellar Purkinje cells [5] and later on classified as a member of the RNase A superfamily [6]. It induces Gordon phenomenon which shows muscle stiffness, ataxia, incoordination, and spasmodic paralysis in animal models [1,7]. EDN shows *in vitro* antiviral activity against RNA viruses, including human respiratory syncytial virus (hRSV), para-influenza virus [8], and human immunodeficiency virus (HIV)-1 [9,10]. Furthermore, recent studies have reported that EDN can be used as a biomarker of eosinophilic esophagitis [11] and amyotrophic lateral sclerosis [12].

EDN and its mouse counterpart, mouse eosinophil-associated RNase 2 (mEAR2), have been reported to act as a selective chemoattractant for dendritic cells (DCs) [13]. They promote activation and maturation of DCs [14] and augment Type 2 helper T cell (Th2)-biased immune responses in a toll-like receptor 2 (TLR2)-dependent manner [15]. TLR2 is expressed on the surface of a wide variety of cells including lung bronchial epithelial cells [16] as well as microglial cells [17] and immune cells, such as DCs and macrophages [18]. Our previous study [19] showed that maltose-binding protein fused EDN (MBP-EDN) could interact with Beas-2B cells, a human bronchial epithelial cell line with limited expression of transcripts of TLR2 gene [16]. It suggested that MBP-EDN might interact with other components (other than TLR2) on cell surface of Beas-2B cells.

EDN shows affinity for heparin, as indicated by its purification in 1986 using heparin-Sepharose column chromatography [20]. We have recently found that heparin oligosaccharides added exogenously inhibit the interaction between EDN and Beas-2B cells [19]. Our data suggested that EDN bound not only heparin used in *in vitro* experiments, but also heparan sulfate (HS) expressed on the surface of Beas-2B cells. Heparin and HS are linear polysaccharides consisting of repeating disaccharide units of α-1,4-linked hexuronic acid and hexosamine [21]. The hexuronic residues typically consist of 90% IdoA and 10% GlcA [22]. Most common disaccharide units of heparin contain 2-*O*-sulfated IdoA and 6-*O*- and *N*-sulfated Glc*N* [23]. In addition to HS, other GAGs such as chondroitin sulfate (CS), dermatan sulfate (DS), and hyaluronic acid (HA) are also present on the cell surface as well as in extracellular matrix [21]. These GAGs have been shown to interact with numerous proteins including cytokines, growth factors, and proteases to modulate functions of proteins, and are implicated in many biological processes including cell growth, development, immunology, and disease processes [24,25].

It is empirically known that heparin binding proteins have domains characterized by the presence of clusters of positively charged residues, such as Arg and Lys, which are likely to promote heparin binding by electrostatic interactions [26]. Two conventional heparin binding sequences, XBBXBX or XBBBXXBX (X is a hydrophobic or uncharged amino acid, and B is a basic amino acid) were classified by sequence comparison of various heparin binding proteins [27]. The amino acid sequence of EDN contains 12 basic amino acids (8 Arg and 4 Lys residues), and nine of them are concentrated within three regions including ^34^QRRCKN^39^ in loop 3, ^65^NKTRKN^70^ in loop 4, and ^113^NRDQRRD^119^ in loop 7 [20]. All of these regions have three basic amino acids in contiguous five residues. Among which the sequence pattern ^34^QRRCKN^39^ matches exactly to the XBBXBX motif [28], and indeed a 10-amino acid peptide, ^32^NYQRRCKNQN^41^, has been demonstrated to be capable of binding heparin [29]. Regarding the other two regions, ^65^NKTRKN^70^ also possesses the XBBXBX pattern in a reverse order, but ^113^NRDQRRD^119^ does not have any known heparin binding sequence. To date, the second and the third regions serving as binding sites for heparin in EDN have not been described.

In this study, the sequences ^34^QRRCKN^39^, ^65^NKTRKN^70^ and ^113^NRDQRRD^119^ were identified as heparin binding regions (HBRs)—*i.e.*, HBR1, HBR2 and HBR3, respectively—and their functional roles in heparin binding were characterized using *in silico* computer modeling and *in vitro* binding assays. Furthermore, the importance of sulfo groups of GAGs in interaction with EDN was characterized.

## 2. Results and Discussion

### 2.1. Binding of MBP-EDN to Heparin and Beas-2B Cells

Neuton D. L. *et al.* [30] have expressed EDN without any tag, and recovered recombinant EDN from inclusion bodies through denaturation, renaturation, dialysis, and repeating purification steps by heparin-Sepharose column and a Sephadex G100 column chromatography. Although untagged, recombinant EDN can be produced by established procedures above; refolding under an artificial condition with low yield makes it time consuming for intensive assay. Producing a protein soluble in host bacteria is a general strategy recombinant protein technology. Thus, to increase protein solubility and recovery yield of recombinant EDN, here we fused MBP tag at *N*-terminus. Nevertheless, potential influence of bulky MBP tag in EDN function by blocking GAG binding sites would be further evaluated.

**Figure 1:**
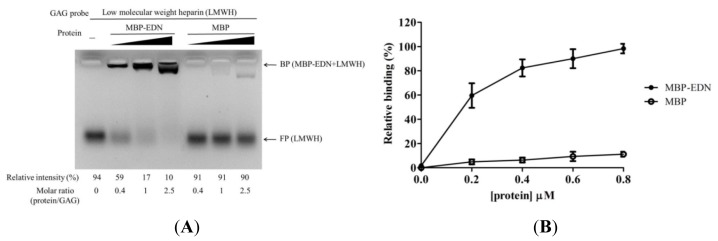
Maltose-binding protein-eosinophil derived neurotoxin (MBP-EDN) binding to low molecular weight heparin (LMWH) and Beas-2B cells. (**A**) 2-aminoacridone (AMAC)-labeled LMWH (0.33 nmol) was incubated with or without MBP-EDN (protein: LMWH ratio of 0.4 to 2.5) in PBS at 25 °C for 15 min, and the reaction products were separated by gel electrophoresis using 1% agarose gel. The probe and protein were indicated at the top of the gel, and the numbers shown at the bottom of the gel indicate the relative intensity (%) of free probe and the molar ratios of protein to LMWH. FP, free probe; BP, bound probe; (**B**) Beas-2B cells were treated with indicated MBP-EDN/MBP concentrations in RPMI 1640 medium at 4 °C for 1 h. The levels of bound proteins were determined by cELISA. The amount of MBP-EDN (a positive control) bound to Beas-2B cells at 0.8 μM was set to 100%, and MBP was used as negative control. The data shown are mean ± SD in triplicate assays.

Binding of recombinant MBP-EDN to heparin and Beas-2B cells was characterized by FACE and cELISA [28]. In the former case, purified MBP-EDN was incubated with 2-aminoacridone-labeled low molecular weight heparin (AMAC-LMWH) at different molar ratios, and the samples were subjected to gel electrophoresis on a 1% agarose gel plate to separate MBP-EDN bound AMAC-LMWH and free polysaccharide. Figure 1A showed that AMAC-LMWH signal shifted upon addition of increasing molar ratio of MBP-EDN over AMAC-LMWH, and 90% signal shifted at a MBP-EDN: AMAC-LMWH ratio of 2.5. In a control experiment, at the same ratio of protein *vs.* AMAC-LMWH, only 10% MBP appeared as a complex with AMAC-LMWH. Concentration-dependent binding of MBP-EDN to Beas-2B cells was also observed by cELISA. MBP showed only a background level of signals (11%), compared to that of MBP-EDN (100%) at 0.8 μM (Figure 1B). These data clearly indicate that binding of MBP-EDN to both LMWH and Beas-2B cells is mediated by the EDN moiety, but not the MBP moiety, of the fusion protein used in this study.

To further differentiate differences in GAG binding affinity between MBP-EDN and refolded recombinant EDN (EDN-6His), heparin binding activity of both was measure as shown in Figure S1. Approximately an 83% shift at 1 molar ratio of protein to glycan suggested that our MBP-EDN (Figure 1A) possessed comparable heparin binding activity to refolded EDN-6His (88% shift) (Figure S1), strongly indicating that MBP tag may not influence ligand binding property of EDN. Our observation was consistent with other studies demonstrating that the MBP tagged proteins such as MBP-tagged eosinophil cationic protein (MBP-ECP) [31] and dengue virus envelope protein [32] still remained GAG binding affinities.

### 2.2. Involvement of Cell Surface GAGs in Binding to MBP-EDN

**Figure 2:**
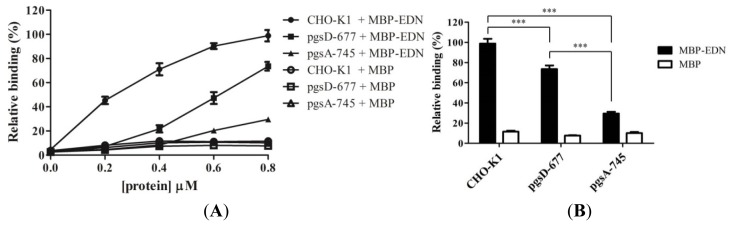
Involvement of glycosaminoglycans (GAGs) on cell surface in binding to MBP-EDN. (**A**) Chinese hamster ovary (CHO)-K1 (control), pgsD-667 (HS-deficient) and pgsA-745 (GAG-deficient) cells were treated with MBP-EDN/MBP at indicated concentrations in Vitacel Ham’s F12K medium supplemented with 10% FBS at 4 °C for 1 h. The amount of 0.8 μM MBP-EDN bound to CHO-K1 cell was set to 100% and MBP was used as a negative control; (**B**) Relative binding activity of each protein at 0.8 μM. The data shown are mean ± SD in triplicate assays. ***, *p* < 0.001.

Interaction of MBP-EDN with cell surface GAGs was further investigated using wild type Chinese hamster ovary (CHO) cell line (CHO-K1) [33], and two mutant cell lines with specific defects in proteoglycan biosynthesis. Both CHO-K1 cells and the two mutant CHO cells showed a dose-dependent binding between 0 and 0.8 μM of MBP-EDN (Figure 2A). Under similar conditions, MBP itself showed negligible levels (less than 10% activity of MBP-EDN) of binding in all three cell lines (Figure 2A).

In the mutant CHO cell line pgsD-677, synthesis of HS is specifically impaired due to lack of HS polymerase that polymerizes disaccharide units of outer HS chains. However, pgsD-677 cells have approximately three times more CS as compared to CHO-K1 cells [34]. The amount of MBP-EDN bound to pgsD-677 cells is reduced by 26% (at 0.8 μM) as compared to that of CHO-K1 cells (Figure 2B), indicating that cell surface HS is at least partially responsible for binding of EDN. The pgsA-745 cell line expresses less than 5% of the GAGs expressed by CHO-K1 cells due to the lack of a xylosyltransferase which catalyzes the first step in biosynthesis of GAG [35]. As shown in Figure 2B, the amounts of MBP-EDN bound to pgsA-745 cells significantly reduced by 71% as compared to CHO-K1 cells. The level of bound MBP-EDN to pgsD-677 cells was significantly reduced than that of pgsA-745 cells, suggesting that cell surface GAGs other than HS may be also involved in the interaction between MBP-EDN and CHO-K1 cells.

**Figure 3:**
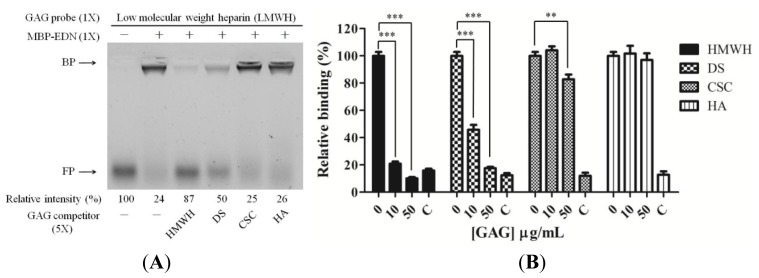
Ligand specificity of major GAGs for interacting with MBP-EDN. (**A**) AMAC-labeled LMWH (0.33 nmol) was pre-incubated with five-fold molar excess of unlabeled GAGs at 25 °C for 5 min, and then incubated with or without MBP-EDN (equal molar ratio) in PBS at 25 °C for 15 min. The reaction products were separated by gel electrophoresis using 1% agarose gel. The amount of AMAC-LMWH without any treatment was set to 100%. Relative intensity (%) of free probe and unlabeled competitors are indicated at the bottom of the gel image. FP, free probe; BP, bound probe; (**B**) Beas-2B cells were pre-incubated with various GAGs in RPMI 1640 medium at 4 °C for 30 min before treatment with 5 μg/mL of MBP-EDN at 4 °C for an additional 1 h. The levels of bound MBP-EDN/MBP were determined by cELISA. The amount of MBP-EDN bound to Beas-2B cells without GAG treatment was set to 100%. C, control. The data shown are mean ± SD in triplicate assays. **, *p*<0.01; ***, *p*<0.001.

To further investigate specificity of MBP-EDN binding, we measured the inhibitory activity of different types of GAGs including high molecular weight heparin (HMWH) which is highly sulfated, chondroitin sulfate C (CSC) and dermatan sulfate (DS) which are moderately sulfated compared to HMWH, and hyaluronic acid (HA). Among these four GAGs, HMWH showed a high level of inhibition (87%) compared with the binding of LMWH to MBP-EDN, and DS reduced LMWH binding by 50% at a GAG: LMWH ratio of 5. However, the same amount of CSC and HA had no effect (Figure 3A). Similar results were obtained by cELISA competition assays (Figure 3B). HMWH and DS significantly reduced MBP-EDN binding to Beas-2B cells in a dose-dependent manner. At a concentration of 10 μg/mL, HMWH and DS reduced MBP-EDN binding to the cells by 79% and 54%, respectively. However, CSC and HA had no effect. At 50 μg/mL, both HMWH and DS showed over 80% of inhibitory effects, whereas CSC manifested only 20% inhibition, and HA did not affect MBP-EDN binding. These data suggest that EDN has a higher affinity to heparin/HS and DS than to other types of GAGs.

HMWH, the most sulfated GAG containing on average 2.7 sulfo groups per disaccharide unit, possessed higher inhibitory activity than DS and CSC that have one sulfo group per disaccharide unit as expected (Figure 3). However, DS and CSC sharing similar sulfation levels showed distinctly different results, indicating that the sugar moieties and other factors were also involved in EDN-GAG interaction. DS has been named as chondroitin sulfate B (CSB) since it has an *N*-acetylgalactosamine (Gal*N*Ac), and is distinguishable from chondroitin sulfate A (CSA) (4-*O*-sulfated) and CSC (6-*O*-sulfated) mainly in the presence of IdoA moiety which also appears in heparin/HS [36]. Since there are many functional similarities between DS and HS, and some GAG binding proteins bind both DS and HS [37], IdoA moiety seems to play an important role in binding specificity for GAG binding protein. The importance of IdoA in hepatocyte growth factor/scatter factor GAG interaction has been shown before [38]. Cell surface GAGs containing IdoA are also required for efficient hRSV infection [39]. These reports support the involvement of the IdoA moiety in EDN-heparin interactions. On the other hand, disaccharide units of both heparin/HS and HA contain Glc*N* in common, in contrast to Gal*N* in DS and CSC. However, the inhibitory activity of HA was very low. These data discount the possibility of the Glc*N* moiety being involved in EDN-GAG interactions.

Many biological activities attributable to EDN and ECP have been characterized and found to be significantly different; nevertheless, EDN and ECP still present similar properties such as ribonucleolytic [40], antiviral [41], and heparin/HS binding activities [28]. Cell surface GAG binding selectivity of ECP has been demonstrated with similar protocol [31]. In addition, binding affinities of ECP to various GAG disaccharides were also predicted by docking simulation [42], showing that ECP had a higher affinity to interact with heparin rather than HA or CSC. Interestingly, HA had a higher inhibitory effect and binding affinity than CSC in these studies, implying that electrostatic interactions were not the only factor involved in EDN/ECP-GAG interactions and the difference in GAG recognition mode between EDN and ECP might arise from diverse biological functions of these two eosinophil RNases.

### 2.3. Requirement of Sulfo Groups on Heparin for Binding to EDN

**Figure 4:**
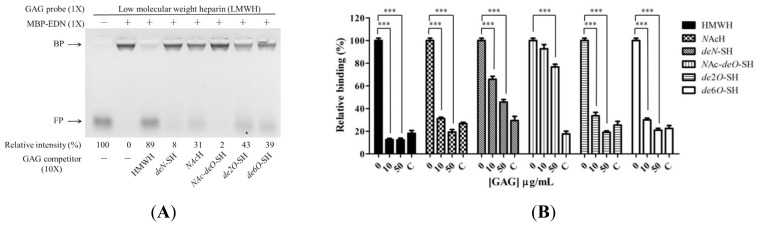
Involvement of heparin sulfo groups in binding to MBP-EDN. (**A**) AMAC-labeled LMWH (0.33 nmol) was incubated with 10-fold molar excess of unlabeled heparin derivatives, individually, prior to incubation with or without MBP-EDN (equal molar ratio) in PBS at 25 °C for 15 min. The reaction products were separated by gel electrophoresis on 1% agarose gels. The amount of AMAC-LMWH without any treatment was set as 100%. Relative intensity (%) of free probe and unlabeled competitors are indicated at the bottom of the gel image. FP, free probe; BP, bound probe; (**B**) Beas-2B cells were pre-incubated with different heparin derivatives in RPMI 1640 medium at 4 °C for 30 min before treatment with 5 μg/mL of MBP-EDN at 4 °C for an additional 1 h. The levels of bound proteins were determined by cELISA, and the amount of MBP-EDN bound to Beas-2B cells pretreatment with no heparin/heparin derivative was set as 100%. C, control. The data shown are mean ± SD in triplicate assays. ***, *p* < 0.001.

The sulfo groups of heparin/HS are important for biological functions including fibroblast growth factor (FGF) signaling [43] and virus infection [44,45]. We tested the effects of sulfo groups of heparin on EDN binding, using intact, untreated HMWH and heparin derivatives: *N-*acetyl heparin (*N*AcH), *de-N*-sulfated heparin (*deN*-SH), *N*-acetyl-*de-O*-sulfated heparin (*N*Ac-*deO*-SH), *de*-2-*O*-sulfated heparin (*de*2*O*-SH), and *de*-6-*O*-sulfated heparin (*de*6*O*-SH). Figure 4A shows that among these compounds HMWH inhibited binding of LMWH to MBP-EDN most effectively (89% inhibition). In contrast, *N*Ac-*deO*-SH (completely devoid of *O*-sulfo group and with a markedly diminished *N*-sulfation) showed the weakest inhibition (2%) of MBP-EDN binding to AMAC-LMWH, indicating that the sulfo groups provided almost all necessary negative charges for binding to EDN. Four other derivatives with selectively modified sulfo groups including *N*AcH, *deN*-SH, *de*2*O*-SH, and *de*6*O*-SH showed 31%, 8%, 43%, and 39% inhibition activities, respectively. Similarly, results of cELISA competition assay revealed that HMWH inhibited 87% of cellular binding of MBP-EDN while *N*Ac-*deO*-SH showed only 7% inhibitory effect at a concentration of 10 μg/mL. Regarding other derivatives, *deN*-SH, *N*AcH, *de*2*O*-SH, and *de*6*O*-SH showed 34%, 69%, 66% and 70% inhibition activities at the same concentration, respectively, compared with the control without competitor. At 50 μg/mL, all heparin derivatives showed more than 50% inhibition whereas *N*Ac-*deO*-SH inhibited only 24% (Figure 4B).

Although heparin lacking of 2-*O*- or 6-*O*-sulfo groups showed a decreased MBP-EDN binding activity, it could not completely abolish the interaction between MBP-EDN and Beas-2B cells. This suggests that EDN might not interact with heparin through only one specific position of sulfo group, unlike FGF-1 or FGF-2, which require 6-*O* sulfated [46] and 2-*O* sulfated heparin [47] for interaction. In addition, the 3-*O*-sulfo group which rarely appears in heparin/HS and has been proposed to be important for interaction with FGF-7 [43] and infection of herpes simplex virus type 1 [44] might also be required for interaction with EDN. With respect to *N*-sulfo groups, although both *N*AcH and *de*-*N*-SH were able to significantly reduce MBP-EDN binding to Beas-2B cells, the inhibitory effect of *N*AcH (69%) was higher than that of *de*-*N*-SH (34%). Besides, out of these two derivatives, only *N*AcH was observed to compete with the interaction between LMWH and MBP-EDN by FACE analysis, suggesting that the *N*-acetyl group of heparin, which appears in most HS sequences, may be responsible for weaker binding to MBP-EDN.

### 2.4. Identification of Critical Basic Residues on EDN for Interaction with Heparin and Other Cell Surface GAG

**Figure 5:**
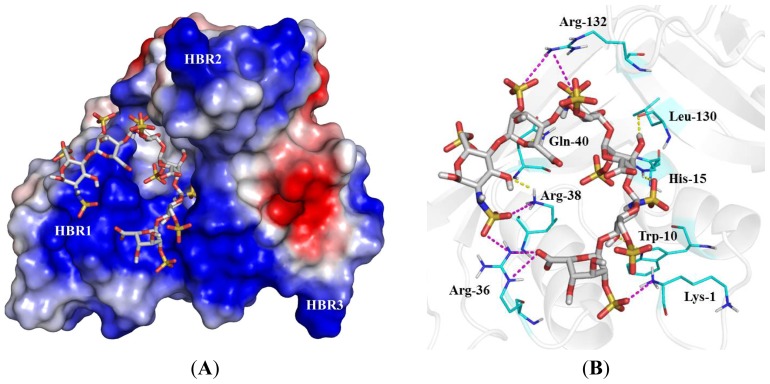
Computer modeling of an EDN-heparin hexasaccharide complex. The stick model is used to represent heparin hexasaccharide. (**A**) Electrostatic potential is mapped onto the solvent-accessible surface of the EDN; a blue color indicates a region of positive potential (+58 kT/e), red indicates a negative potential (−58 kT/e), and white indicates a neutral potential; (**B**) Binding mode of heparin hexasaccharide to EDN with the protein shown in cartoon representation. Selected amino acid side chains of EDN are shown in line representation. The ionic interactions between EDN and heparin are shown in violet dashed lines and hydrogen bonds are shown in yellow dashed lines.

Minimal length of heparin molecule required for inhibition of MBP-EDN/ECP cellular binding activity has been demonstrated [19,28]. With increasing oligosaccharide length, the degree of MBP-EDN/ECP cellular binding decreased and pentasaccharide served as the minimal length to inhibit cellular binding activities of these proteins. Since 90% of cellular binding activity of EDN was inhibited by 10 μg/mL heptasaccharide [19], we proposed that heptasaccharide was required for efficient interaction with MBP-EDN. It should be noted that heptasaccharide contained a matrix sugar at the reducing end in the study, suggesting that heparin hexasaccharide might be a suitable length for exerting molecular interaction between EDN and heparin molecule. Thus, heparin hexasaccharide was used to perform computer modeling for EDN-heparin hexasaccharide complex structure and identification of potential binding poses and residues on EDN involved in heparin binding. The molecular surface of EDN shows a V-shaped cleft. This cleft contains positively charged residues (Figure 5A). HBR1 is one of the surface regions that constitute the cleft, and a part of HBR2 is located on the edge of the cleft. HBR3 is not a part of the cleft but it was located on the same side of the molecular surface as HBR1 and HBR2. Our model suggests that the bound heparin hexasaccharide was stabilized by ionic interactions with Arg^36^ and Lys^38^ in HBR1, and Lys^1^ and Arg^132^ which did not belong to HBRs, whereas Arg^97^ and basic residues in HBR2 and HBR3 were not located within van der Waals force/hydrogen bond distances to interact with the heparin hexasaccharide (Figure 5B). Other than electrostatic interactions, heparin hexasaccharide also showed potential interactions with EDN by mainly van der Waals (vDW) force and six hydrogen bonds between sugar backbone and Lys^1^, Trp^10^, His^15^, Arg^36^, Gln^40^ and Leu^130^ as shown in Figure 5B and Table S1.

**Figure 6:**
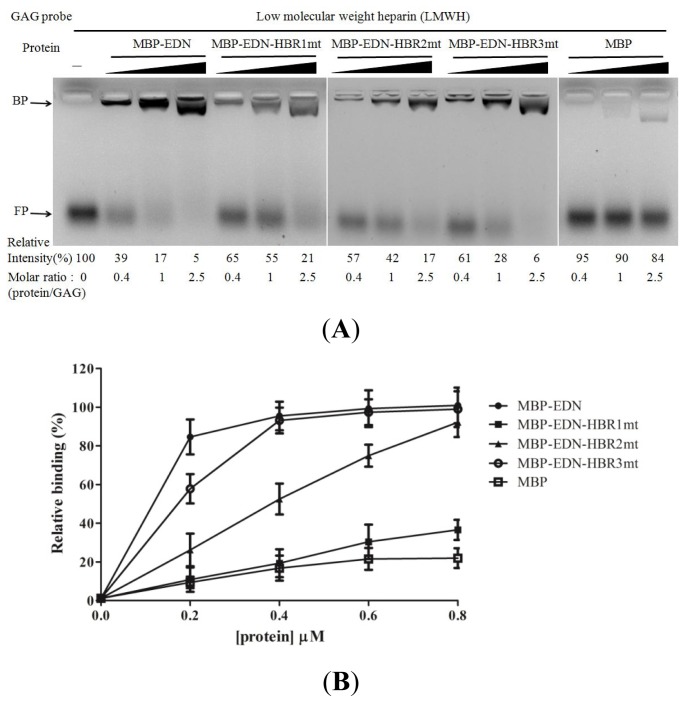
Involvement of HBRs on MBP-EDN binding to LMWH and Beas-2B cells. (**A**) AMAC-labeled LMWH (0.33 nmol) was incubated with each HBR-mutated MBP-EDN (protein: LMWH ratio of 0.4 to 2.5) in PBS at 25 °C for 15 min, and the reaction products were separated by gel electrophoresis on 1% agarose gels. Probes and proteins used are indicated at the top of the gel. The amount of AMAC-LMWH without any treatment was set as 100%. The numbers shown at the bottom of the gel images indicate the relative intensity (%) of free probe and the molar ratios of proteins to LMWH. MBP was used as a negative control. FP, free probe; BP, bound probe; (**B**) Beas-2B cells were separately treated with 0 μM to 0.8 μM HBR-mutated MBP-EDN. The levels of bound proteins were determined by cELISA. The amount of MBP-EDN bound to Beas-2B cells at 0.8 μM was set as 100%. The data shown are mean ± SD of triplicate assays.

MBP-EDN-HBR mutants, in which the basic residues in HBRs were replaced with Ala, were generated to investigate the functional roles of HBRs in EDN. All mutants showed a weaker interaction with AMAC-LMWH at different molar ratios of protein/GAG (Figure 6A). The intensity of bound AMAC-LMWH in MBP-EDN, MBP-EDN-HBR1mt (R35A/R36A/K38A), MBP-EDN-HBR2mt (K66A/R68A/K69A), MBP-EDN-HBR3mt (R114A/R117A/R118A), and MBP at equimolar ratios with respect to LMWH were 83%, 45%, 58%, 72%, and 10%, respectively. MBP-EDN-HBR1mt at a concentration of 0.2 μM also showed only 11% cellular binding activity, indicating that HBR1, ^34^QRRCKN^39^, was a key heparin binding motif in EDN. Regarding MBP-EDN-HBR2mt and MBP-EDN-HBR3mt, although their cellular binding activity was reduced to respectively 26% and 58% at 0.2 μM with increasing protein concentration, the cellular binding activity was comparable to that of MBP-EDN (Figure 6B). This suggests that ^65^NKTRKN^70^ (HBR2) and ^113^NRDQRRD^119^ (HBR3) play auxiliary roles in the interaction between MBP-EDN and GAGs. Natural heparin is a heterogeneous mixture with molecular weights ranging from 5000 Da to over 40,000 Da (HMWH). LMWHs, in contrast, consist of only short chains of heparin of average molecular weight of less than 8000 Da (approximately 10 disaccharide units). The basic residues of HBR2 and HBR3 which are too far to interact with the heparin hexasaccharide in the computer model might act as a secondary heparin binding sites on EDN and bind with much lower affinity to long chain heparin molecules. Recently, the loop L7 of EDN containing HBR3 has been shown to play an important role in anti-hRSV activity of EDN [48]. Since hRSV infection and hRSV vaccines elicit neutralizing and non-neutralizing antibodies reactive with envelope glycoproteins [49], EDN might recognize specific glycan structures on the virus envelope. Each HBR might be involved in different EDN functions through target selectivity.

Putative HBRs on ECP including ^34^RWRCK^38^, ^73^RSRFR^77^, and ^101^RPGRR^105^ have been aligned with the corresponding segments of the other 12 RNases employing Clustal W2 [50]. Among these three HBRs, only HBR1 on ECP (^34^RWRCK^38^) is found in a position comparable with HBR1 on EDN (^34^QRRCK^38^) within a primary sequence that is 60% homologous. However, all the HBRs on EDN and ECP were located at a similar position and surrounded by an electropositive cavity in their tertiary structures. Peptide containing HBR1 in ECP (NYRWRCKNQNK) had similar LMWH binding activity to the corresponding region of EDN (NYQRRCKNQNK) in FACE analysis [28]. However, mutant MBP-ECPmt1 (R34A/W35A/R36A/K38A) showed only 50% cellular binding activity as compared to wild type protein at 20 μg/mL (0.4 μM) [28]. Here, mutant MBP-EDN-HBR1mt (R35A/R36A/K38A) showed only 20% at the same concentration. These results indicated that the main cellular binding activity of EDN lies in the primary sequence#HBR1, whereas ECP may interact with cell surface GAGs through not only HBR1 but also other basic residues (Arg^1^, Arg^7^ and His^64^) and polar residues (Gln^14^, Asn^39^ and Gln^40^) surrounded in a basic cavity as prediction model illustrated in a previous study [42].

**Figure 7:**
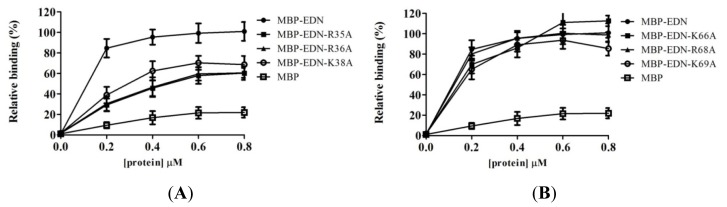

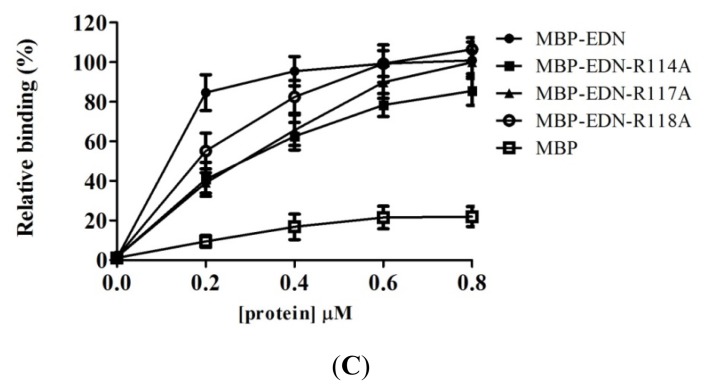
Involvement of single basic residues on MBP-EDN in binding to Beas-2B cells. Beas-2B cells were separately incubated with various MBP-EDN with a single point mutation. The level of bound protein was determined by ELISA. (**A**) R35A, R36A, or K38A; (**B**) K66A, R68A, or K69A; and (**C**) R114A, R117A, or R118A. The amount of MBP-EDN bound to Beas-2B cells at 0.8 μM was set as 100% and MBP was used as a negative control. The data shown are mean ± SD in triplicate assays.

To identify the key residues of the three HBRs of EDN essential for binding to Beas-2B cells, we generated MBP-EDN single point mutants, in which each basic residue within the HBRs was individually changed into Ala. Among all the single mutants, the cellular binding activities of those with R35A, R36A, K38A, K69A, R114A, R117A, and R118A replacements were significantly reduced to 29%, 31%, 39%, 65%, 41%, 39%, and 55%, respectively, as compared to the control at protein concentration of 0.2 μM. Instead, mutations of Lys^66^ and Arg^68^ resulted in little or insignificant effects (Figure 7 and Table 1). In contrast, K1A, R97A and R132A mutants had no effect (data not shown). These results suggested that the basic residues within HBRs play more important roles in GAG binding and Arg^35^, Arg^36^, and Arg^38^ in HBR1 and Arg^114^ and Arg^117^ in HBR3 are particularly important for the interaction.

ECP structure in complex with heparin disaccharide [51] and trisaccharide heparin mimetic [52] have been previously resolved by NMR. The results indicate the crucial role of basic cavity on ECP surface in heparin binding and point out the contribution of residues surrounding the cavity. Other than basic residues, aromatic residue, Trp^35^, and several polar residues including Gln^14^, Asn^39^, and Gln^40^ are also involved in these complexes. As for EDN, although MBP-EDN-HBR1mt (R35A/R36A/K38A) showed major contribution in cellular binding, it still possessed 45% binding activity to LMWH in FACE analysis. In addition, there are six hydrogen bonds in our EDN-heparin hexasaccharide prediction model, revealing that contribution of other aromatic and polar residues such as His, Trp, Asn and Gln should be taken into consideration.

## 3. Experimental Section

### 3.1. Antibodies and Reagents

Monoclonal mouse anti-MBP was purchased from Santa Cruz (Dallas, TX, USA) and goat anti-mouse horseradish peroxidase (HRP) was purchased from Jackson ImmunoResearch (West Grove, PA, USA). Low molecular weight heparin (LMWH), high molecular weight heparin (HMWH; heparin sodium salt), dermatan sulfate (DS), chondroitin sulfate C (CSC), hyaluronic acid (HA), *N-*acetyl heparin (*N*AcH), *de-N*-sulfated heparin (*deN*-SH), and *N*-acetyl-*de-O*-sulfated heparin (*N*Ac-*deO*-SH) were purchased from Sigma-Aldrich (St. Louis, MO, USA). *De*-2-*O*-sulfated heparin (*de*2*O*-SH), and *de*-6-*O*-sulfated heparin (*de*6*O*-SH) were purchased from Neoparin (Alameda, CA, USA). Chemicals were purchased from Sigma-Aldrich (St. Louis, MO, USA) unless otherwise specified.

### 3.2. Cells and Cell Culture

Beas-2B (ATCC number: CRL-9609), a human bronchial epithelial cell line, wild type Chinese hamster ovary (CHO)-K1 (ATCC number: CRL-9618), mutant CHO cell lines pgsD-677 (ATCC number: CRL-2244) and pgsA-745 (ATCC number: CRL-2242) were purchased from American Type Culture Collection (Manassas, VA, USA). Beas-2B cells (ATCC number: CRL-9609) were cultured in RPMI 1640 medium (Sigma-Aldrich, St. Louis, MO, USA) supplemented with heat-inactivated 10% (*v*/*v*) fetal bovine serum (FBS) (Gibco/Invitrogen, Grand Island, NY, USA) and 1% (*v*/*v*) Penicillin, Streptomycin, and Amphotericin (Biosera, Kansas City, MO, USA). CHO cells were cultured in Vitacel Ham’s F12K medium (Sigma-Aldrich, St. Louis, MO, USA) supplemented with 10% (*v*/*v*) FBS.

### 3.3. Construction, Expression and Purification of MBP-EDN Mutants

The DNA encoding EDN without the signal sequence was amplified using PCR primers 5′-GAATTCAAACCTCCACAGTTTACC-3′ and 5′-TCTAGATTAGATGATTCTATCCAG-3′ and cloned into the pMAL-c2G plasmid (New England Biolabs, Hitchin, UK) between the *Eco*RI and *Xba*I sites to generate pMAL-c2G-*edn*. The plasmid was used as a template to generate basic amino acid single mutant including R35A, R36A, K38A, K66A, R68A, K69A, R114A, R117A and R118A using QuikChange site-directed mutagenesis (Stratagene, Santa Clara, CA, USA). In addition, these residues were simultaneously substituted to Ala to generate the following three mutants: MBP-EDN-HBRmt1 (R35A/R36A/K38A), MBP-EDN-HBRmt2 (K66A/R68A/K69A), and MBP-EDN-HBRmt3 (R114A/R117A/R118A) using the same protocol. To express a wild type MBP-EDN and its mutants, the plasmid was used to transform *Escherichia coli* (*E. coli*) BL21 (DE3) Gold cells (Stratagene, Santa Clara, CA, USA). Two milliliters of the overnight culture was inoculated in 100 mL LB broth containing 100 μg/mL carbencillin, and incubated at 37 °C until OD_600_ reached 0.4–0.6. IPTG (Ameresco, Solon, OH, USA) was added to a final concentration of 0.5 mM, and incubated at 30 °C for 4 h. After protein induction, the bacteria were harvested by centrifugation for 10 min at 2900× *g*, and the pellet was homogenized by EmulsiFlex-C3 (AVESTIN, Ottawa, ON, Canada). Wild type MBP-EDN and all mutant proteins in the soluble portion of bacterial cell lysates were purified by amylose affinity chromatography (New England Biolab, Hitchin, UK).

### 3.4. Construction, Expression and Purification of Recombinant EDN Containing a C-Terminal 6His-Tag (EDN-6His)

The *edn* gene was cloned into the pET23a plasmid (New England Biolabs, Hitchin, UK) between *Xba*I and *Bam*HI to generate pET23a-*edn*. The expression procedures were same as MBP-EDN but misfolded EDN-6His was accumulated in inclusion bodies. Recombinant EDN-6His was purified by Ni^2+^ Sepharose™ (GE Healthcare, Uppsala, Sweden) and refolded and dialyzed as previously described [53].

### 3.5. Cell-Based Enzyme-Link Immunosorbent Assay (cELISA)

Confluent monolayers of Beas-2B or CHO cells in 96-well plate were incubated with different concentrations of MBP-EDN at 4 °C for 60 min. The cells were washed with 200 μL of ice-cold PBS, and fixed with 2% paraformaldehyde (PFA)/PBS at 25 °C for 15 min. Then, 200 μL of ice-cold PBS was added to wash the cells prior to blocking with 2% BSA/PBS at 25 °C for 90 min. The level of bound MBP-EDN was quantified by ELISA analysis using a mouse monoclonal anti-MBP IgG and a HRP-conjugated goat anti-mouse IgG as the primary and secondary antibody, respectively. After the cells were washed by ice-cold PBS, 50 μL of Super Signal^®^ West Pico Chemiluminescent Substrate (Thermo, Waltham, MA, USA) was added and the chemiluminescent intensity was measured by Wallace Vector II (LS55, PerkinElmer, Santa Clara, CA, USA). The amount of MBP-EDN bound to cells without heparin derivatives or GAGs treatment was set to 100%.

### 3.6. Fluorescence-Assisted Carbohydrate Electrophoresis (FACE)

GAGs were labeled with 2-aminoacridone (AMAC) according to the reported procedures [54]. Briefly, 50 μg of each GAG was incubated with 40 μL of a solution containing 1.25 M AMAC, 85% DMSO, and 15% acetic acid at 25 °C for 15 min. To this, 40 μL of 1.25 M sodium cyanoborohydride (NaBH_3_CN) was added, and the mixture was incubated at 37 °C for 16 h. At the end of the reaction, 720 μL of 95% ice-cold ethanol was added and rested for 15 min at −20 °C. The sample was centrifuged at 4 °C for 5 min at 14,000× *g*. The supernatant was carefully discarded, and the pellet was freeze-dried by ScanVac CoolSafe (LaboGene™, Lynge, Denmark). The dried pellet was dissolved in sterile deionized water at an appropriate concentration for a measurement. The AMAC-labeled probes thus prepared were stored at −80 °C until use.

AMAC-labeled probes and proteins were mixed and incubated at 25 °C for 15 min. The mixture was loaded onto a 1% agarose gel plate, and electrophoresed for 20 min in a buffer containing 40 mM Tris-acetic acid, 1 mM EDTA, pH 8.0. The AMAC labeled probe was detected under UV light and the gel was scanned by QUANTUM-ST4 (Vilber Lourmat, Eberhardzell, Germany).

### 3.7. Binding Competition Assay

For competition assay, monolayers of Beas-2B cells in 96-well plates were pre-incubated with serially diluted heparin derivatives or GAGs in serum-free RPMI 1640 medium at 4 °C for 30 min. To the samples, MBP-EDN was added, and incubated at 4 °C for 60 min. Bound MBP-EDN was measured as described above.

### 3.8. Computer Modeling of EDN-Heparin Oligosaccharide Complex

The structure of EDN was taken from PDB database (PDB entry 1HI2) [55], and energy minimized with the GROMOS 96 force field. *In silico* docking experiments were performed using AutoDock Vina software (AutoDock, La Jolla, CA, USA) [56]. Preliminary docking runs were performed with comprising three of ^1^C_4_-IdoA(2S)–Glc*N*S(6S). In order to find preferred side chain conformations, all basic residues in three HBRs of EDN were set to be flexible in computation. Global search was performed in the grid box with a dimension of 60 × 60 × 60 angstroms that covered the entire protein. For computer simulation, we chose heparin hexasaccharide with chair forms of IdoAs as a ligand and used predetermined coordinates for the side chains of basic residues within HBRs.

### 3.9. Statistical Analysis

Statistical analysis was performed using GraphPad Prism 5 (GraphPad Software, La Jolla, CA, USA). All data are shown in mean ± standard deviation (SD). Statistical significance between a set of data was determined by unpaired two-tailed Student’s *t*-test.

## 4. Conclusions

In conclusion, membrane GAGs, especially HS and DS, were essential for EDN interaction with Beas-2B cells. The Sulfo group of heparin, predominantly 2-*O*- or 6-*O*-sulfo group, is an obligatory factor for EDN interaction to heparin and Beas-2B cells. A combination of *in silico* bioinformatics analyses and *in vitro* functional assays has successfully identified that basic residues Arg^35^, Arg^36^, and Arg^38^ in HBR1, and Arg^114^ and Arg^117^ in HBR3 of EDN play a critical role in GAG binding. These results provide an insight into the molecular interaction of EDN with soluble heparin and bronchial epithelial cells which may have biomedical implications for the physiological functions of eosinophil RNases.

## Supplementary Information



## Figures and Tables

**Table 1:** Beas-2B cell binding activity of MBP-EDN wild type and mutants.

Protein (0.2 μM)	Mean ± SD (%)
MBP-EDN	84.6 ± 9.0
R35A	28.5 ± 5.8 ***
R36A	31.0 ± 7.0 ***
K38A	38.9 ± 8.0 ***
K66A	69.9 ± 8.8 *
R68A	80.1 ± 6.5
K69A	65.1 ± 9.8 ***
R114A	40.8 ± 8.5 ***
R117A	39.0 ± 5.1 ***
R118A	55.1 ± 9.0 ***
MBP-EDN-HBR1mt	10.9 ± 6.3 ***
MBP-EDN-HBR2mt	26.2 ± 8.4 ***
MBP-EDN-HBR3mt	57.8 ± 7.6 ***
MBP	9.4 ± 3.0

The average amount of 0.8 μM MBP-EDN bound to Beas-2B cells was set as 100% binding.

* *p* < 0.05;

*** *p* < 0.001.

## Abbreviations

AMAC: 2-aminoacridone
CHO: Chinese hamster ovary
CS: chondroitin sulfate
CSA: chondroitin sulfate A
CSB: chondroitin sulfate B
CSC: chondroitin sulfate C
DC: dendritic cell
DS: dermatan sulfate
*de*2*O*-SH: *de*-2-*O*-sulfated heparin
*de*6*O*-SH: *de*-6-*O*-sulfated heparin
*deN*-SH: *de-N*-sulfated heparin
ECP: eosinophil cationic protein
EDN: eosinophil derived neurotoxin
FACE: fluorescence-assisted carbohydrate electrophoresis
FGF: fibroblast growth factor
GAG: glycosaminoglycan
HS: heparan sulfate
HBR: heparin binding region
HMWH: high molecular weight heparin
HRP: horseradish peroxidase
HIV: human immunodeficiency virus
hRSV: human respiratory syncytial virus
HA: hyaluronic acid
LMWH: low molecular weight heparin
MBP: Maltose-binding protein
*N*Ac-*deO*-SH: *N*-acetyl-*de-O*-sulfated heparin
TLR2: toll-like receptor 2
*N*AcH: *N-*acetyl heparin
PFA: paraformaldehyde
SD: standard deviation
mEAR: mouse eosinophil-associated RNase
Th2: Type 2 helper T cell
