# Supplementary material for: Basic Amino Acid Residues of Human Eosinophil Derived Neurotoxin Essential for Glycosaminoglycan Binding
Source: Int J Mol Sci. 2013 Sep 16;14(9):19067–85. doi: 10.3390/ijms140919067 (PMC3794821; doi:10.3390/ijms140919067)
Supplement: Supplementary file 1 [file ijms-14-19067-s001.pdf]

Supplementary Information

Table S1. Predicted interaction between wild type EDN and heparin hexasaccharide

**Residue**	**Atom/group**	**Type of interaction**	**Residue**	**Atom/group**
Lys[1]	α-NH_3^+	H-bond/Ionic	IDS6	Oxygen of 2-*O*-sulfate
Trp[7]	C-4	vDW	SGN5	Oxygen of *N*-sulfate
	C-5	vDW	SGN5	C-1
Trp[10]	N-1	H-bond	SGN5	Oxygen of 3-OH
Gln[14]	Amide NH_2	vDW	IDS4	Oxygen of 2-*O*-sulfate
	Amide NH_2	vDW	SGN5	Nitrogen, hydrogen, and oxygen of *N*-sulfonyl group
His[15]	Hydrogen of τ-nitrogen	H-bond	SGN5	Oxygen of *N*-sulfonyl group
	C-2	vDW	IDS4	Oxygen of 2-*O*-sulfate
Gln[34]	Carbon of side-chain amide group	vDW	IDS6	Ring oxygen
Arg[36]	δ- and ω-Nitrogen	H-bond/Ionic	IDS6	Oxygen of C-6 carboxyl group
	ω-Nitrogen	Ionic	SGN1	Oxygen of *N*-Sulfonyl group
	C-5	vDW	SGN1	Oxygen of *N*-Sulfonyl group
Lys[38]	ε-NH_3^+ group	Ionic	SGN1	Oxygen of *N*-Sulfonyl group
	Hydrogen of ε-NH_3^+ group	vDW	SGN1	Oxygen of C3-OH group
Asn[39]	Hydrogen of side-chain amide group	vDW	SGN1	Oxygen of sulfonyl group
Gln[40]	C-3	vDW	IDS2	C-1
	C-4	vDW	IDS2	C-1 and oxygen of 2-*O*-sulfonyl group
	Nitrogen of side-chain amide group	vDW	IDS2	C-1, C-5, and C-6
	Nitrogen of side-chain amide group	H-bond	IDS2	Ring oxygen
His[129]	C-5	vDW	IDS4	C-4
	C-5	vDW	SGN5	C-1
Leu[130]	C-1	vDW	IDS4	Hydrogen of C-3-OH
	Backbone carbonyl oxygen	H-bond	IDS4	C-3-OH
Arg[132]	ω-Nitrogen	Ionic	SGN3	*N*-sulfate
	ω-Nitrogen	Ionic	IDS2	2-*O*-sulfate

H-bond, hydrogen bond; vDW, van der Waals force; SGN, 6-*O*-sulfated, *N*-sulfated glucosamine; IDS, 2-*O*-sulfated iduronic acid.

Figure S1. Binding activity of recombinant EDN-6His to low molecular weight heparin (LMWH). FACE was carried out by incubating 2-aminoacridone (AMAC)-labeled LMWH (0.33 nmol) with or without increasing concentrations of proteins in PBS at 25 °C for 15 min, and the reaction products were separated on a 1% agarose gel. The reacted probe and protein were shown above the gel, and the numbers below the gel indicated the relative intensity (%) of free probe and the molar ratio of protein to LMWH. FP, free probe; BP, bound probe.

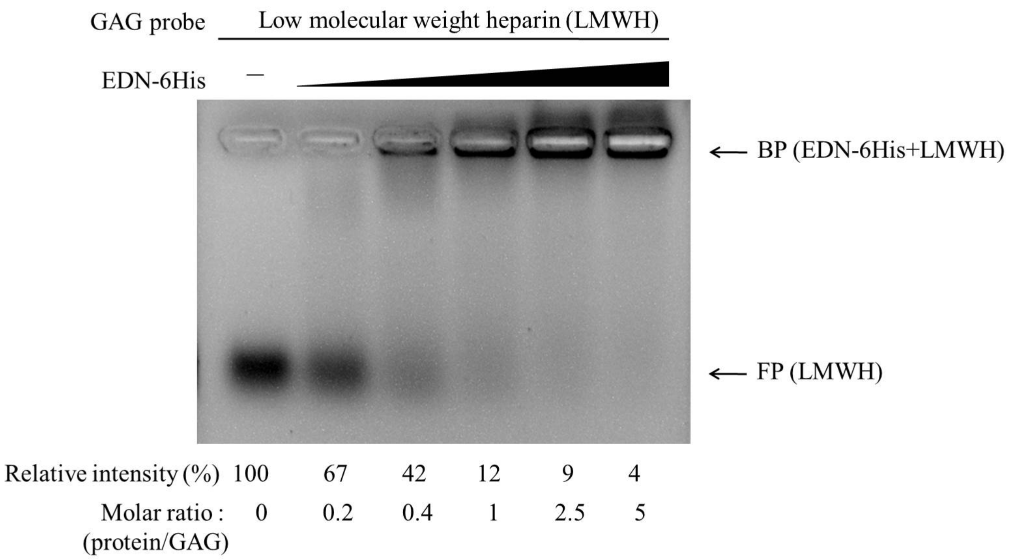

© 2013 by the authors; licensee MDPI, Basel, Switzerland. This article is an open access article distributed under the terms and conditions of the Creative Commons Attribution license (http://creativecommons.org/licenses/by/3.0/).
